# The HIV care cascade: a systematic review of data sources, methodology and comparability

**DOI:** 10.7448/IAS.18.1.20634

**Published:** 2015-11-30

**Authors:** Nicholas A Medland, James H McMahon, Eric PF Chow, Julian H Elliott, Jennifer F Hoy, Christopher K Fairley

**Affiliations:** 1 Central Clinical School, Monash University, Melbourne, VIC, Australia; 2 Melbourne Sexual Health Centre, Alfred Health, Melbourne, VIC, Australia; 3 Department of Infectious Diseases, The Alfred Hospital and Monash University, Melbourne, VIC, Australia

**Keywords:** HIV, cascade, treatment as prevention, antiretroviral therapy, treatment coverage, population-based data, HIV care cascade, HIV treatment cascade

## Abstract

**Introduction:**

The cascade of HIV diagnosis, care and treatment (HIV care cascade) is increasingly used to direct and evaluate interventions to increase population antiretroviral therapy (ART) coverage, a key component of treatment as prevention. The ability to compare cascades over time, sub-population, jurisdiction or country is important. However, differences in data sources and methodology used to construct the HIV care cascade might limit its comparability and ultimately its utility. Our aim was to review systematically the different methods used to estimate and report the HIV care cascade and their comparability.

**Methods:**

A search of published and unpublished literature through March 2015 was conducted. Cascades that reported the continuum of care from diagnosis to virological suppression in a demographically definable population were included. Data sources and methods of measurement or estimation were extracted. We defined the most comparable cascade elements as those that directly measured diagnosis or care from a population-based data set.

**Results and discussions:**

Thirteen reports were included after screening 1631 records. The undiagnosed HIV-infected population was reported in seven cascades, each of which used different data sets and methods and could not be considered to be comparable. All 13 used mandatory HIV diagnosis notification systems to measure the diagnosed population. Population-based data sets, derived from clinical data or mandatory reporting of CD4 cell counts and viral load tests from all individuals, were used in 6 of 12 cascades reporting linkage, 6 of 13 reporting retention, 3 of 11 reporting ART and 6 of 13 cascades reporting virological suppression. Cascades with access to population-based data sets were able to directly measure cascade elements and are therefore comparable over time, place and sub-population. Other data sources and methods are less comparable.

**Conclusions:**

To ensure comparability, countries wishing to accurately measure the cascade should utilize complete population-based data sets from clinical data from elements of a centralized healthcare setting, where available, or mandatory CD4 cell count and viral load test result reporting. Additionally, virological suppression should be presented both as percentage of diagnosed and percentage of estimated total HIV-infected population, until methods to calculate the latter have been standardized.

## Introduction

According to UNAIDS Executive Director Michel Sidibé, we have the tools at our disposal to reduce HIV incidence as well as AIDS-related morbidity and mortality [1]. The UNAIDS global HIV targets of 90% diagnosed, 90% on antiretroviral treatment and 90% suppressed suggest that testing and antiretroviral therapy (ART) are these tools. The format of these targets mirrors the cascade of HIV diagnosis, care and treatment (HIV care cascade), placing it at the very centre of the global HIV response [2]. Resource-rich and resource-limited countries alike are looking to the HIV care cascade to guide and measure interventions to achieve high ART coverage [3, 4].

ART is now thought to have a clinical benefit to all individuals living with HIV, including those with asymptomatic infection and high CD4 count [5, 6]. However, ART also has an effect beyond the individual clinical benefit. Reduction in transmission at both individual and population levels with ART has now been established [7, 8].

The HIV care cascade is a tool to determine what proportion of the HIV-infected population enjoys the clinical and epidemiological benefits of virological suppression, where interventions to improve coverage of clinical care should occur and how their success might be measured. Accurate and reproducible measurement of the cascade will be necessary to assess progress in regard to stated goals and to successfully develop and implement interventions to improve the cascade. The association between HIV incidence and ART coverage is the principle underlying treatment as prevention. How this works in practice in real world populations can only be investigated when both incidence and coverage are measured consistently.

The HIV care cascade typically combines different measurements and estimations of important data elements from HIV infection through to HIV viral load suppression. The first step in the cascade is the number of individuals living with HIV infection and the second step is the number living with diagnosed HIV infection. Linkage (or initial engagement in care) and retention (or recent engagement in care), ART and virological suppression are either estimated or measured and reported as a number or a proportion of an earlier step in the cascade [9].

Although comparisons of the cascade between countries or jurisdictions, over time and within sub-populations are illuminating in understanding the obstacles to universal ART coverage [10, 11], we wondered how variations in data sources and methodology affected the comparability of data. The objective of this review was to examine the data sources and methodology used in available cascades of HIV diagnosis, care and treatment and to assess their comparability.

## Methods

**Figure 1:**
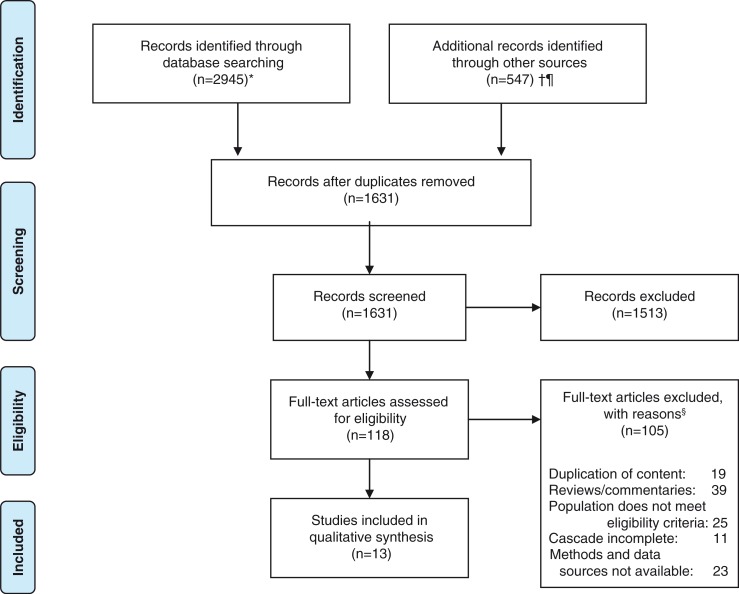
Study selection. ^1^Published and unpublished literature were searched using the string HIV AND (cascade OR continuum); *PubMed, Medline (Ovid), CINAHL (Ebscohost); †unpublished literature: a. conference abstracts: CROI 2015, AIDS 2014, CROI 2014, CROI 2013, IAS 2013, HIV Drug Therapy Conference (Glasgow) 2014, b. specific websites (UNAID, WHO, Government Websites of OECD member countries) [14]. Authors were contacted to provide the complete paper where it was not available; ^¶^additional records identified from search of reference lists; ^§^some studies had more than one reason for exclusion.

We performed a systematic search of published literature up to 15 March 2015 using three electronic journal databases – PubMed, Medline (via Ovid) and CINAHL (via Ebscohost) – using the search string “HIV” AND (“continuum” OR “cascade”). Additional records were identified by searching HIV/AIDS, government and conference websites (listed in Figure 1) that were considered unpublished documents using the same search string. Only English language sources were searched. Screening was performed on full abstracts for published literature and on title and abstract, where available, for unpublished literature. Full text articles were downloaded from databases for assessment of eligibility. Where the full text article was not available, the author was contacted by email and invited to submit the document. Screening and assessment were performed by NAM and independently verified by JHM and EPFC.

We applied the following eligibility criteria: first, a cascade had to contain at least three elements and include the element *population diagnosed with HIV* and either *ART* or *virological suppression*; second, the cascade had to apply to a population that could be defined geographically, demographically or by risk group association but not by enrolment in a healthcare service or system or study; and third the data sources and methods had to be described or cited for each element of the cascade.

We examined the following cascade elements: the total number of people with HIV (including those not diagnosed), individuals who had been diagnosed with HIV infection, linkage (or initial engagement in care), retention (or recent engagement in care), prescription or receipt of ART and virological suppression.

We used a standardized, piloted data extraction form to collect the following data: definition of cascade element, data sources, method of estimation or calculation and result. Data collection was performed by NAM.

Eligible studies are referred to as *cascades* and are described by the year from which the data were derived (not the year of publication), the jurisdiction and sub-population.

Data sources for each step of the cascade, except the undiagnosed or total population, were categorized as follows: complete population-based data (individual-level data collected across the whole population, as that population is defined by the cascade); partial population-based data (individual-level data collected across part of the population, as that population is defined by the cascade); sample data (representative samples where sampling had been performed with the prior intention of producing representative data for this purpose); or studies (studies, samples or cohorts that were not necessarily designed to be generalizable for the specific purpose of generating these data).

We defined as most-comparable those cascade elements using population-based data collected from the entire population (complete). Less comparable were those cascade elements using different population-based data sets from across part of the population (for example if population-based data were only available from part of the jurisdiction, from certain time periods or from certain subsets of the population), from representative samples (which might be comparable to other cascades using the same method) or from non-representative samples (which would not be reproduced or reproducible in other cascades).

This review protocol was registered with PROSPERO (No. CRD42015016718) and reported in accordance with the PRISMA (Preferred Reporting Items for Systematic Reviews and Meta-Analyses) statement [12, 13].

## Results and discussion

A total of 3492 records were identified through database search and other resources. After removal of duplicate records, we screened 1631 records, of which 118 reports were selected for review of the full text article and 13 were identified as eligible for inclusion (Figure 1).

Seven cascades were published by the US Centers for Disease Control and Prevention (CDC). The CDC 2008 cascade, covering care in the 1.18 million people living with HIV in the United States in 2008, was published in 2011 [15], whereas the CDC 2009 cascade was published in 2013 [16] and the CDC 2011 cascade in 2014 [17]. The CDC 2010 men who have sex with men (MSM) cascade [18], the CDC 2010 blacks cascade [19] and the CDC 2010 Hispanics cascade[20] covered the 416,730 MSM, the 353,653 blacks and the 172,536 Hispanics and Latinos living with diagnosed HIV infection in 2010 in the United States, respectively, all published in 2014. The CDC 19-jurisdiction 2010 cascade reported on the 338,959 people living with diagnosed HIV in 2010 in the 19 states and jurisdictions with mandatory reporting of CD4 and viral load [21].

Also within the United States, the New York City 2010 cascade [22] and the King County (Washington State) 2011 cascade [23], both published in 2014, covered the 87,146 people living with diagnosed HIV in New York City in 2010 and the 6094 people living with HIV in King County in 2011, respectively.

Outside of the United States, the British Columbia 2011 cascade [24] reported on the 13,140 people living with HIV in that Canadian province. The Denmark 2010 cascade reported on the 10,136 patients living with diagnosed HIV and the Georgia 2012 cascade on all 4900 people living with HIV in those countries [25, 26]; the Australia 2014 cascade [27] reported on the 26,000 people living with HIV in that country in 2014.

### Data sources

Of the 13 cascades, seven included the total number of individuals infected with HIV (including those not diagnosed), all 13 reported the number who had been diagnosed with HIV infection, 12 reported linkage to care, all 13 reported retention in care, 11 reported ART and all 13 reported virological suppression (Table 1).

**Table 1:** Data sources and reported rate of virological suppression

Cascade	Living with HIV	Diagnosed	Linked to care	Retained in care	Receive ART	Suppression	Suppression (% total)	Suppression (% diagnosed)
CDC 2008 US [15]	As cited^a^ [28]	Partial^b^	Studies [29, 30]	Studies [29–32]	Sample^c^	Sample^c^	28	35
CDC 2009 US [16]	As cited^a^ [33, 34]	Partial^b^	Partial^d^	Sample^c^	Sample^c^	Sample^c^	25	31
CDC 2010 US MSM [18]	–	Partial^b^	Partial^d^	Partial^d^	Sample^c^	Sample^c^	–	42
CDC 2010 US blacks [19]	–	Partial^b^	Partial^d^	Partial^d^	Sample^c^	Sample^c^	–	35
CDC 2010 US Hispanics [20]	–	Partial^b^	Partial^d^	Partial^d^	Sample^c^	Sample^c^	–	37
CDC 2010 US jurisdictions [21]	–	Complete^e^	Complete^f^	Complete^f^	–	Complete^f^	–	43
CDC 2011 US [17]	As cited^a^ [35]	Complete^e^	Partial^d^	Sample^c^	Sample^c^	Sample^c^	30	35
New York City 2010 [22]	–	Complete^e^	–	Complete^f^	–	Complete^f^	–	59
King County 2011 [23]	As cited^a^ [36]	Complete^e^	Complete^g^	Complete^g^	Sample^h^	Complete^g^	57	67
British Columbia 2011 [24]	As cited^a^ [37]	Complete^e^	Complete^i^	Complete^i^	Complete^i^	Complete^i^	35	49
Australia 2014 [27]	As cited [38, 39]	Complete^e^	Complete^f^	Studies [40]	Studies [40]	Studies [40]	62	72
Denmark 2010 [25]	–	Complete^e^	Complete^i^	Complete^i^	Complete^i^	Complete^i^	–	70
Georgia 2012 [26]	As cited^a^ [41]	Complete^e^	Complete^i^	Complete^i^	Complete^i^	Complete^i^	20	39

a Back-calculation and/or other methods using data sources and methods as indicated in citation;

b complete data available from part of the jurisdiction. Transition from AIDS notification to HIV notification available from different jurisdictions for different time periods;

c the Medical Monitoring Project is a US CDC study of healthcare delivery based on a national representative sample [34];

d complete data available from part of the jurisdiction: CD4 and viral load data from those parts of the jurisdiction with mandatory reporting;

e centrally managed registry of mandatory notification of HIV diagnoses;

f centrally managed registry of mandatory notification of CD4 and viral load data;

g centrally managed registry of mandatory notification of CD4 and viral load data plus other methods;

h local Medical Monitoring Project data plus population based chart reviews;

i clinical data from a centralized linked clinical care database; CDC, Centers for Disease Control and Prevention; MSM, men who have sex with men; ART, antiretroviral therapy.

Of the seven cascades that reported an estimate of the of the total population living with HIV, including undiagnosed infection, each cited a different back-calculation method and data sources (Table 1).

We observed three types of population-based data sets used in the measurement of the remaining cascade elements. First, centrally maintained registers of mandatory or statutory reporting of HIV diagnosis provided data for measurement of the population with diagnosed HIV. Second, centrally maintained registers of mandatory or statutory reporting of CD4 count and viral load test results provided a population-based data set for measuring linkage into care, retention in care and virological suppression. For linkage and retention, the date of the test marks a clinical episode that is used to indicate engagement in care at that time. For virological suppression, the value of the viral load test result is used. Third, population-based clinical data on all patients in the jurisdiction were available in those jurisdictions that had linked clinical databases and a linked electronic health record as components of a centrally managed healthcare system.

Table 1 shows the data sources used by each cascade.

All cascades used the same methodology and data source to report diagnosed HIV: a national HIV surveillance system. However, only 8 of the 13 (62%) cascades had data available from the whole population (i.e. complete population-based data set). Six of twelve cascades (50%) reporting linkage to care used population-based data from the whole population. Six of thirteen (46%) cascades reporting retention in care, 3 of 11 (28%) reporting ART and 6 of 13 (46%) reporting virological suppression used population-based data from the entire population.

### Definitions

All cascades defined the diagnosed population as those alive, resident in the jurisdiction and having received a diagnosis. Twelve cascades reported linkage to care, of which 11 (92%) defined it as evidence of medical care (engagement) within three months of diagnosis. Twelve of thirteen (92%) defined *retention* as evidence of engagement in care within the most recent year. Additionally, nine cascades included retention in continuous care and defined it as two or more episodes in the most recent year separated by at least three months. Eleven cascades (84%) reported ART, of which seven defined it as drug prescription (64%) and 4 (36%) as dispensed drug. Eight of thirteen cascades (62%) defined *suppression* as most recent viral load below a cutoff of 200 copies/ml, three (23%) used 400 copies/ml and one cascade each used 500 copies/ml and 50 copies/ml. Table 2 shows the cascade element definitions used by each cascade.

**Table 2:** Definitions of cascade elements

Cascade	Living with HIV	Diagnosed	Linked to care*	Retained in care**	ART	Suppression (copies/ml)
CDC 2008 US [15]	Back-calculation	Notified	Cited studies	Cited studies	Prescribed	<200
CDC 2009 US [16]	Back-calculation	Notified	Three months^a^	One year^a^	Prescribed	<200
CDC 2010 US MSM [18]	–	Notified	Three months^a^^,^ c	One year^c^	Prescribed	<200
CDC 2010 US blacks [19]	–	Notified	Three months^a^^,^ c	One year^c^	Prescribed	<200
CDC 2010 US Hispanics [20]	–	Notified	Three months^a^^,^ c	One year^c^	Prescribed	<200
CDC 2010 US jurisdictions [21]	–	Notified	Three months^a^^,^ c	One year^c^	–	<200
CDC 2011 US [17]	Back-calculation	Notified	Three months^a^	One year^a^	Prescribed	<200
New York City 2010 [22]	–	Notified^d^	–	One year^c^	–	<200
King County 2011 [23]	Back-calculation	Notified^e^	Three months^c^	One year^c^	Dispensed	<400
British Columbia 2011 [24]	Back-calculation	Notified	Three months^a^	One year^a^	Dispensed	<50
Australia 2014 [27]	Back-calculation	Notified^f^	Three months^c^	One year^c^	Prescribed	<400
Denmark 2010 [25]	–	Notified^g^	Three months or ever^a^	One year^a^	Dispensed	<400
Georgia 2012 [26]	Back-calculation	Notified	Three months or ever^a^	One year^a^	Dispensed	<400

* *Linkage* is defined as evidence of care provision within the time period specified after HIV diagnosis.

** *Retention* is defined as evidence of care provision within the time interval specific before the study time point;

a linkage in patients diagnosed in the final year before the study point was applied to the whole population;

b clinical care episode – any recorded clinical event;

c clinical care episode inferred from date of a reported viral load or CD4 count result;

d cases for whom no data were available in the most recent five years were assumed to have left the jurisdiction or died [42];

e cases for whom no data were available in the most recent year were investigated;

f adjustment of 8% duplication for notifications [43];

g adjustment for known international migration.

### Discussion

This is the first review of the methodology of the HIV care cascade, and substantial differences were found in the data sources and methods used to calculate the elements of the cascade, limiting their comparability. Those cascade elements considered most comparable were diagnosed HIV, retention in care and virological suppression. Cascades that used complete population-based data sets in all cascade elements, with the exclusion of undiagnosed HIV, came from states and countries with mandatory reporting of CD4 count and viral load test results or clinical data derived from linked clinical databases covering the entire population.

## Living with HIV

No complete measure exists for the estimate of the total population living with HIV, including those who have never been tested, making this cascade element the most difficult to assess. An example of the difficulty in back-calculations is highlighted by the Australian cascade, which used three prevalence surveys and two methods of back-calculation to calculate “plausible” ranges from 11.1 to 21.2% for undiagnosed infection using different sources and methods [27]. Each cascade included in this review used different data sources and different methods to calculate this cascade element, limiting their comparability.

Population treatment coverage is commonly expressed as the proportion of the estimated total population living with HIV infection with virological suppression. However, uncertainty as to the size of the denominator has a downstream effect on suppression rates and limits their comparability to other settings.

Many cascades do not include the estimated population living with HIV, including those who have not been diagnosed. Although this has the practical benefit of eliminating a major source of uncertainty, it also undermines the value of the cascade overall by removing the impact of diagnosis and testing on the rates of virological suppression. Using consistently available data in the same way might improve comparability at least. For example, a novel method developed by Jansson *et al*. [39] estimates incidence and prevalence, making use of the date and value of first-recorded CD4 count.

## Diagnosed with HIV

Although all cascades used a centralized notification of diagnosis of HIV infection, parts of the United States transitioned at different times from a notification system based on clinical AIDS diagnosis to one based on a diagnosis of HIV infection. As a result, complete population-based data sets on number of individuals living with diagnosed HIV infection were not available from all states and all time periods for some US cascades [15, 16, 18–20]. This factor limits the comparability of the results to other cascades that did not have this restriction.

Importantly, individuals who have died, moved away or were incorrectly notified will not appear in current patient registers and may appear to have been lost to care. A method to remove these individuals from the denominator is required to avoid underestimating retention in care. Examples of such a method include the study from the King County health department, where staff individually investigated every case where reported laboratory results had ceased [44]. They found that 1018 of 5123 individuals diagnosed with HIV in King County had moved away. The NYC cascade, using a method described by Dombrowski, excluded an unspecified number of individuals who had not accessed care in the previous five years from the number living with HIV, assuming that they had died or moved away [42], whereas the Danish cascade accessed passport control records to account for individuals who had left the country [25].

In the King County method, mandatory reporting of viral load and CD4 count allows identification of individuals who are lost to care or who have died or moved away, so that they can either be encouraged to return to care or removed from the denominator [23]. Collecting and acting on these data, therefore, has the potential to affect individual and population care outcomes as well as improving the validity of the cascade.

Jurisdictions whose surveillance/notification systems do not account for incorrect notification or undocumented migration or death will overestimate the size of the population living with diagnosed HIV and underestimate the rates of treatment coverage and virological suppression by an unknown amount. Observed differences between jurisdictions, over time and between sub-populations will be difficult to interpret. As a result, public health policy and interventions based on these figures have the potential to be misdirected and their outcomes difficult to evaluate.

## Linkage and retention in care

Two types of population-based data were used by cascades included in this review to measure linkage and retention in care. First, clinical care databases where all individuals in care in that jurisdiction were receiving care in a single centralized healthcare system or a system using the same clinical care database were used in the Danish, British Columbian and Georgian cascades [24–26]. Second, viral load test and CD4 count results were available in jurisdictions with mandatory reporting of those data and were used in the King County, New York City and CDC 19 jurisdiction cascades [21–23].

The British Columbian, Danish and Georgian cascades had access to clinical care data as a component of a centrally managed healthcare system. Clinical care databases allow direct measurement of care indicators for patients in those jurisdictions. These measurements include records of clinical care episodes which do not result in a viral load test or CD4 count such as prescribing or dispensing ART, or adherence counseling. These data can be used to precisely determine linkage and retention in care, as well as ART.

However, in many countries patients receive care in a variety of settings where sharing or pooling clinical data is not feasible. In these places reporting of viral load and CD4 count can allow us to accurately determine the rate of virological suppression and to make close estimates of engagement and linkage to care.

## Treatment and suppression

Only British Columbia, Denmark and Georgia were able to access population-based data that included ART. These jurisdictions have access to clinical data from linked and shared clinical databases, which are a component of their centrally managed healthcare system. All other cascades used less comparable data sets.

## Limitations

The currently published cascades over-represent wealthier countries. No eligible cascades were available from low income countries, where the HIV burden is larger and fewer resources are available for reporting of data and HIV diagnosis, care and treatment.

We chose a conservative categorization of data sources. Although very high quality samples, surveys and studies may well approach the reproducibility of complete data sets, it remains difficult to establish their comparability.

We restricted our analysis to the effect of different data sources on the comparability of cascades. Differences in definitions of cascade elements also affect results, as demonstrated by Nosyk and colleagues, who applied different definitions to the data set included in the British Columbian cascade [24].

## Conclusions

The number of cascades published is increasing substantially. Jurisdictions are increasingly analyzing and presenting their data in the cascade format, with 11 of the 13 cascades published in 2014 or 2015.

Differences in the data sources used to estimate or measure the steps in the cascade limit the ability to compare coverage of diagnosis, care, treatment and suppression to other jurisdictions, other time periods or sub-populations. Consequently, where differences between cascades are observed, one cannot say with certainty that those differences reflect a difference in coverage, as opposed to a difference in the data source.

**Figure 2:**
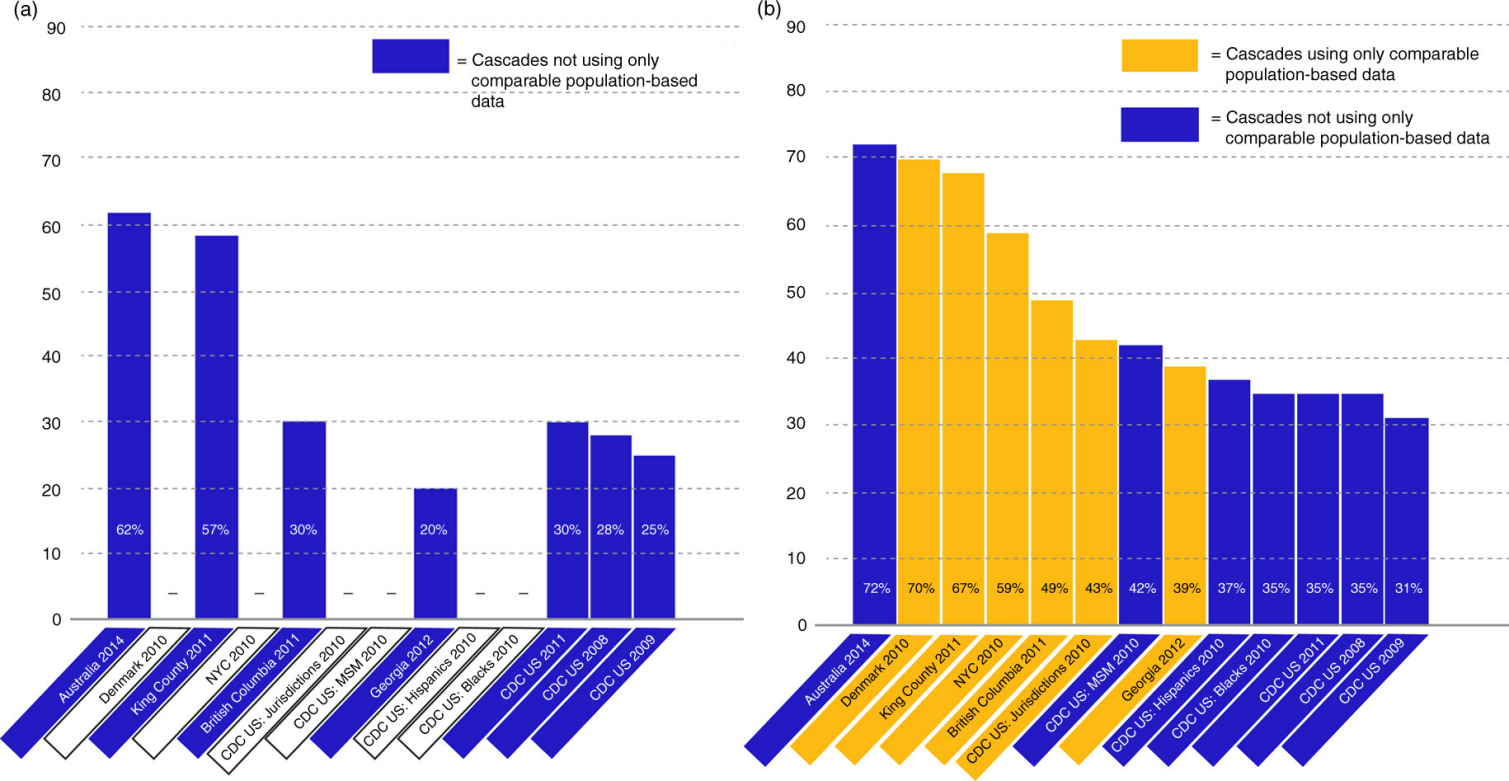
**(a) Rate (%) of virological suppression in estimated total populations of people living with HIV, including undiagnosed infection.** Cascades reporting rate of virological suppression in the estimated population living with HIV, including undiagnosed infection, are shown. No cascades used comparable population-based data in these calculations. **(b) Rate (%) of virological suppression in populations of people living with diagnosed HIV.** Cascades reporting rate of virological suppression in the population living with diagnosed HIV are shown. The lighter bars indicate cascades that use comparable, population-based data. These results can be considered to be comparable. The darker bars indicate cascades using less comparable data. The results of cascades indicated with the darker bars cannot be considered to be comparable.

Considering together the reported rate of virological suppression for all cascades included in this review as a proportion of the estimated total population living with HIV, including undiagnosed HIV infection (Figure 2a) and as a proportion of the population with diagnosed HIV (Figure 2b), it is clear that very few of these results are derived entirely from comparable data sources. Some cascades appear to have higher or lower rates of suppression than others. However, where the data sources are not comparable, conclusions cannot be drawn from this observation.

For example, while it might appear from such a comparison that some jurisdictions have a lower rate of virological suppression than other jurisdictions represented, another explanation is that the available data limit their ability to ensure that undocumented deaths, migration or repeat notification in parts of the country with different HIV notification systems do not inflate the denominator.

Moreover, whereas some cascades report high rates of virological suppression, the complex methodology imposed on the cascade by the available data limits the conclusions that can be drawn from this or from comparisons to other cascades.

A proposed standardized cascade, based on comparable data sources and commonly used or available definitions, is presented in Table 3. We propose using population-based data for all steps in the cascade, including as inputs for back-calculation of the total population living with HIV. All data, except ART, could be derived from HIV notifications and clinical data or mandatory CD4 and VL results. It is recommended that methods to account for undocumented death, migration and multiple testing be employed consistently in all future cascades. It is recognized that implementation of these methods may be unfeasible outside smaller programs in wealthier jurisdictions.

**Table 3:** Proposed recommended standard cascade format

Cascade element	Data source	Definitions, methods
Infected	HIV notifications plus clinical linked individual data or mandatory CD4 cell count and viral load	Back-calculation based on complete data sets*
Diagnosed	HIV notifications	Investigation for undetected migration or death
Linked	Clinical linked individual data or mandatory CD4 cell count and viral load	Within three months of diagnosis
Retained	Clinical linked individual data or mandatory CD4 cell count and viral load	One episode in the most recent year
On treatment	Clinical linked individual data or representative sample	Dispensed or prescribed
Suppressed	Clinical linked individual data or mandatory CD4 cell count and viral load	Viral load <200 copies/ml

* For example Jansson *et al*. [39] proposed back-calculation based on complete data set of CD4 count.

The resulting highly comparable HIV care cascade could be used to measure progress to universal treatment coverage and the success of treatment as prevention, at least in those settings where such measures can be implemented. These measurements would be highly comparable when repeated over time in the same jurisdiction and also comparable across different jurisdictions. In addition we recommend that, until back-calculations are standardized, virological suppression be presented both as a percentage of diagnosed and a percentage of estimated total infected population.

Comparability of virological suppression over time and place are dependent on data sources and methods of estimating cascade elements. Countries wishing to accurately measure the cascade and to explore or exploit the benefits of treatment as prevention should utilize elements of a centralized healthcare setting, where available, or mandatory CD4 and viral load reporting.

## Supplementary Material

The HIV care cascade: a systematic review of data sources, methodology and comparability

The HIV care cascade: a systematic review of data sources, methodology and comparability

## References

[1] Sidibe M, Zuniga JM, Montaner J (2014). Leveraging HIV treatment to end AIDS, stop new HIV infections, and avoid the cost of inaction. Clin Infect Dis.

[2] UNAIDS (2014). 90-90-90 an ambitious treatment target to help end the HIV epidemic.

[3] Obama B Executive order – HIV care continuum initiative 2013 [Internet].

[4] NSW Health NSW HIV Strategy 2012–2015 2014 Data report [Internet].

[5] TEMPRANO Study Group (2015). A trial of early antiretrovirals and isoniazid preventive therapy in Africa. N Engl J Med.

[6] INSIGHT START Study Group (2015). Initiation of antiretroviral therapy in early asymptomatic HIV infection. N Engl J Med.

[7] Cohen MS, Chen YQ, McCauley M, Gamble T, Hosseinipour MC, Kumarasamy N (2011). Prevention of HIV-1 infection with early antiretroviral therapy. N Engl J Med.

[8] Tanser F, Barnighausen T, Grapsa E, Zaidi J, Newell ML (2013). High coverage of ART associated with decline in risk of HIV acquisition in rural KwaZulu-Natal, South Africa. Science.

[9] Gardner EM, McLees MP, Steiner JF, Del Rio C, Burman WJ (2011). The spectrum of engagement in HIV care and its relevance to test-and-treat strategies for prevention of HIV infection. Clin Infect Dis.

[10] Nachega JB, Uthman OA, del Rio C, Mugavero MJ, Rees H, Mills EJ (2014). Addressing the Achilles’ heel in the HIV care continuum for the success of a test-and-treat strategy to achieve an AIDS-free generation. Clin Infect Dis.

[11] Raymond A, Hill A, Pozniak A (2014). Large disparities in HIV treatment cascades between eight European and high-income countries – analysis of break points. J Int AIDS Soc.

[12] Medland NA, Fairley CK, Elliott JH, Chow EPF, McMahon JH The cascade of HIV diagnosis, care and treatment: a systematic review of methodology and comparability. PROSPERO 2015: CRD42015016718.

[13] Liberati A, Altman DG, Tetzlaff J, Mulrow C, Gotzsche PC, Ioannidis JP (2009). The PRISMA statement for reporting systematic reviews and meta-analyses of studies that evaluate health care interventions: explanation and elaboration. PLoS Med.

[14] OECD Member Countries [Internet] http://data.worldbank.org/about/country-and-lending-groups%20-%20OECD_members.

[15] Cohen SM, Van Handel MM, Branson BM, Blair JM, Hall HI, Hu X (2011). Vital signs: HIV prevention through care and treatment – United States. MMWR Morb Mortal Wkly Rep.

[16] Hall HI, Frazier EL, Rhodes P, Holtgrave DR, Furlow-Parmley C, Tang T (2013). Differences in human immunodeficiency virus care and treatment among subpopulations in the United States. JAMA Intern Med.

[17] Bradley H, Hall HI, Wolitski RJ, Van Handel MM, Stone AE, LaFlam M (2014). Vital Signs: HIV diagnosis, care, and treatment among persons living with HIV – United States, 2011. MMWR Morb Mortal Wkly Rep.

[18] Singh S, Bradley H, Hu X, Skarbinski J, Hall HI, Lansky A (2014). Men living with diagnosed HIV who have sex with men: progress along the continuum of HIV care – United States, 2010. MMWR Morb Mortal Wkly Rep.

[19] Whiteside YO, Cohen SM, Bradley H, Skarbinski J, Hall HI, Lansky A (2014). Progress along the continuum of HIV care among blacks with diagnosed HIV – United States, 2010. MMWR Morb Mortal Wkly Rep.

[20] Gant Z, Bradley H, Hu X, Skarbinski J, Hall HI, Lansky A (2014). Hispanics or Latinos living with diagnosed HIV: progress along the continuum of HIV care – United States, 2010. MMWR Morb Mortal Wkly Rep.

[21] Gray KM, Cohen SM, Hu X, Li J, Mermin J, Hall HI (2014). Jurisdiction level differences in HIV diagnosis, retention in care, and viral suppression in the United States. J Acquir Immune Defic Syndr.

[22] Torian LV, Xia Q, Wiewel EW (2014). Retention in care and viral suppression among persons living with HIV/AIDS in New York City, 2006–2010. Am J Public Health.

[23] Dombrowski JC, Buskin SE, Bennett A, Thiede H, Golden MR (2014). Use of multiple data sources and individual case investigation to refine surveillance-based estimates of the HIV care continuum. J Acquir Immune Defic Syndr.

[24] Nosyk B, Montaner JS, Colley G, Lima VD, Chan K, Heath K (2014). The cascade of HIV care in British Columbia, Canada, 1996–2011: a population-based retrospective cohort study. Lancet Infect Dis.

[25] Helleberg M, Haggblom A, Sonnerborg A, Obel N (2013). HIV care in the Swedish-Danish HIV cohort 1995–2010, closing the gaps. PLoS One.

[26] Chkhartishvili N, Sharavdze L, Chokoshvili O, DeHovitz JA, del Rio C, Tsertsvadze T (2015). The cascade of care in the Eastern European country of Georgia. HIV Med.

[27] The Kirby Institute (2014). HIV, viral heptatitis and sexually transmissive infection in Australia Annual Surveillance Report 2014 HIV supplement.

[28] CDC (2011). HIV surveillance – United States, 1981–2008. MMWR Morb Mortal Wkly Rep.

[29] Marks G, Gardner LI, Craw J, Crepaz N (2010). Entry and retention in medical care among HIV-diagnosed persons: a meta-analysis. AIDS.

[30] Torian LV, Wiewel EW (2011). Continuity of HIV-related medical care, New York City, 2005–2009: do patients who initiate care stay in care?. AIDS Patient Care STDS.

[31] Hall HI, Mahle KC, Tang T, Li J, Johnson AS, Shouse L (2011). Retention in care of HIV-infected adults and adolescents in 13 U.S. areas.

[32] Tripathi A, Youmans E, Gibson JJ, Duffus WA (2011). The impact of retention in early HIV medical care on viro-immunological parameters and survival: a statewide study. AIDS Res Hum Retroviruses.

[33] Centers for Disease Control and Prevention (2012). Diagnoses of HIV infection and AIDS in the United States and dependent areas, 2010.

[34] Hall HI, Song R, Rhodes P, Prejean J, An Q, Lee LM (2008). Estimation of HIV incidence in the United States. JAMA.

[35] CDC (2014). Monitoring selected national HIV prevention and care objectives by using HIV surveillance data – United States and 6 dependent areas – 2012.

[36] Chen M, Rhodes PH, Hall IH, Kilmarx PH, Branson BM, Valleroy LA (2012). Prevalence of undiagnosed HIV infection among persons aged >/=13 years – National HIV Surveillance System, United States, 2005–2008. MMWR Morb Mortal Wkly Rep.

[37] Boulos D, Yan P, Remis RS, Archibald CP (2006). Estimates of HIV prevalence and incidence in Canada, 2005. Can Commun Dis Rep.

[38] Jansson J, Kerr CC, Wilson DP (2014). Predicting the population impact of increased HIV testing and treatment in Australia. Sex Health.

[39] Jansson J, Kerr CC, Mallitt K, Wu J, Gray RT, Wilson DP (2015). Inferring HIV incidence from case surveillance with CD4 counts. In review. AIDS.

[40] The Kirby Institute Australian HIV Observational Database Annual Report.

[41] UNAIDS Spectrum EPP 2013 [Internet].

[42] Dombrowski JC, Kent JB, Buskin SE, Stekler JD, Golden MR (2012). Population-based metrics for the timing of HIV diagnosis, engagement in HIV care, and virologic suppression. AIDS.

[43] Law MG, McDonald AM, Kaldor JM (1996). Estimation of cumulative HIV incidence in Australia, based on national case reporting. Aust NZJ Public Health.

[44] Buskin SE, Kent JB, Dombrowski JC, Golden MR (2014). Migration distorts surveillance estimates of engagement in care: results of public health investigations of persons who appear to be out of HIV care. Sex Transm Dis.

