# Supplementary material for: The HIV care cascade: a systematic review of data sources, methodology and comparability
Source: J Int AIDS Soc. 2015 Nov 30;18(1):20634. doi: 10.7448/IAS.18.1.20634 (PMC4666907; doi:10.7448/IAS.18.1.20634)
Supplement: The HIV care cascade: a systematic review of data sources, methodology and comparability [file JIAS-18-20634-s001.pdf]

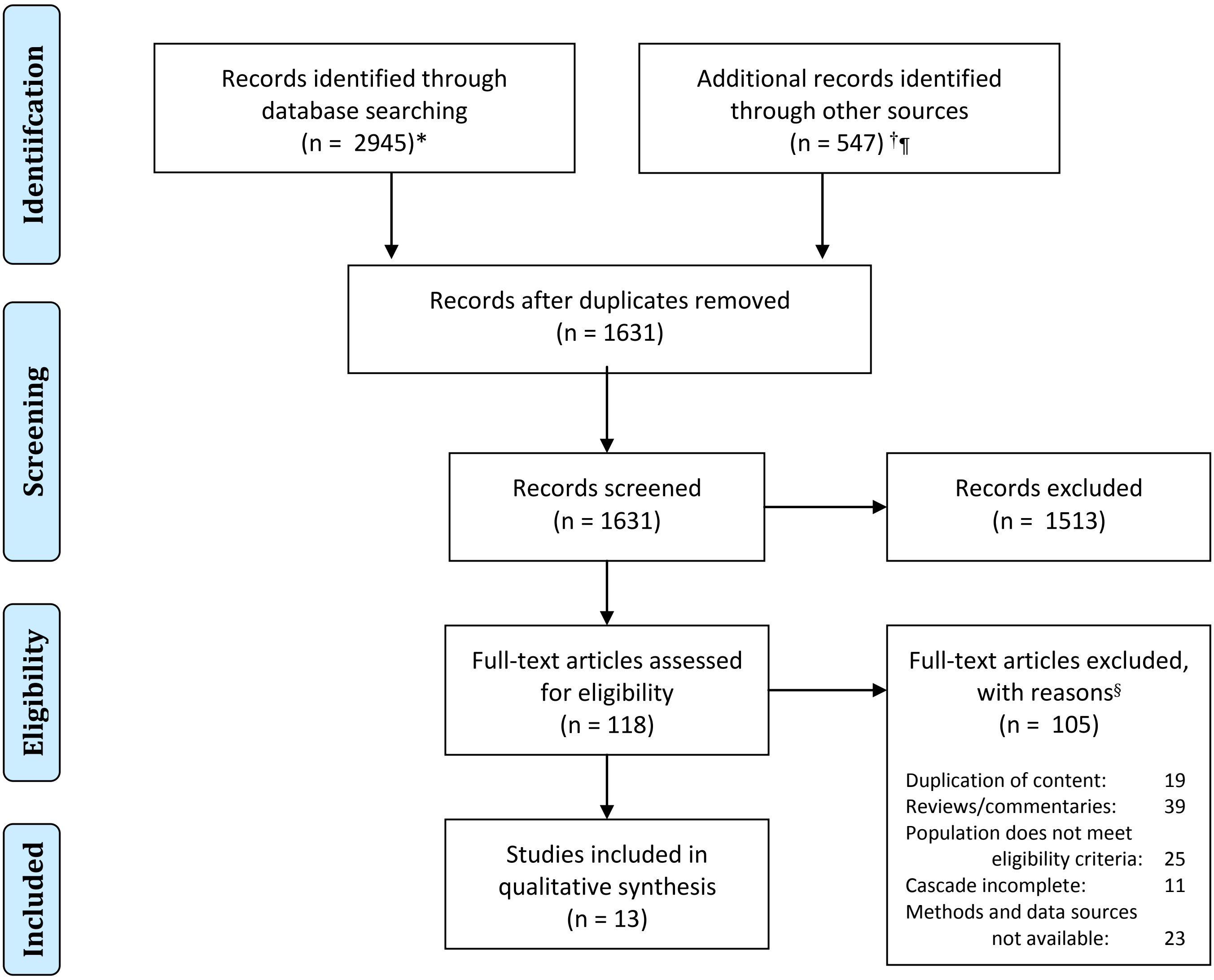

Notes:
1.published and unpublished literature was searched using the string (HIV AND (cascade OR continuum)
* PubMed, Medline (Ovid), CINAHL (Ebscohost)
†Unpublished literature: a. conference abstracts: CROI 2015, AIDS 2014, CROI 2014, CROI 2013, IAS 2013, HIV Drug Therapy Conference (Glasgow) 2014, b. specific websites (UNAID, WHO, Government Websites of OECD member countries)[14]. Authors were contacted to provide the complete paper where it was not available.
¶Additional records identified from search of reference lists
§Some studies had more than one reason for exclusion.

Figure 1. Study Selection
